# Update of the FANTOM web resource: enhancement for studying noncoding genomes

**DOI:** 10.1093/nar/gkae1047

**Published:** 2024-11-27

**Authors:** Tomoe Nobusada, Chi Wai Yip, Saumya Agrawal, Jessica Severin, Imad Abugessaisa, Akira Hasegawa, Chung Chau Hon, Satoru Ide, Masaru Koido, Atsushi Kondo, Hiroshi Masuya, Shinya Oki, Michihira Tagami, Toyoyuki Takada, Chikashi Terao, Nishad Thalhath, Scott Walker, Kayoko Yasuzawa, Jay W Shin, Michiel J L de Hoon, Piero Carninci, Hideya Kawaji, Takeya Kasukawa

**Affiliations:** RIKEN Center for Integrative Medical Sciences, Yokohama, Kanagawa 230-0045, Japan; RIKEN Center for Integrative Medical Sciences, Yokohama, Kanagawa 230-0045, Japan; RIKEN Center for Integrative Medical Sciences, Yokohama, Kanagawa 230-0045, Japan; RIKEN Center for Integrative Medical Sciences, Yokohama, Kanagawa 230-0045, Japan; RIKEN Center for Integrative Medical Sciences, Yokohama, Kanagawa 230-0045, Japan; RIKEN Center for Integrative Medical Sciences, Yokohama, Kanagawa 230-0045, Japan; RIKEN Center for Integrative Medical Sciences, Yokohama, Kanagawa 230-0045, Japan; Tokyo Metropolitan Institute of Medical Science, Tokyo 156-8506, Japan; RIKEN Center for Integrative Medical Sciences, Yokohama, Kanagawa 230-0045, Japan; Graduate School of Frontier Sciences, The University of Tokyo, Tokyo 277-0882, Japan; RIKEN Center for Integrative Medical Sciences, Yokohama, Kanagawa 230-0045, Japan; RIKEN BioResource Research Center, Tsukuba, Ibaraki 305-0074, Japan; Kumamoto University, Kumamoto 860-0811, Japan; RIKEN Center for Integrative Medical Sciences, Yokohama, Kanagawa 230-0045, Japan; RIKEN BioResource Research Center, Tsukuba, Ibaraki 305-0074, Japan; RIKEN Center for Integrative Medical Sciences, Yokohama, Kanagawa 230-0045, Japan; Clinical Research Center, Shizuoka General Hospital, Shizuoka 420-8527, Japan; RIKEN Center for Integrative Medical Sciences, Yokohama, Kanagawa 230-0045, Japan; RIKEN Center for Integrative Medical Sciences, Yokohama, Kanagawa 230-0045, Japan; RIKEN Center for Integrative Medical Sciences, Yokohama, Kanagawa 230-0045, Japan; RIKEN Center for Integrative Medical Sciences, Yokohama, Kanagawa 230-0045, Japan; RIKEN Center for Integrative Medical Sciences, Yokohama, Kanagawa 230-0045, Japan; RIKEN Center for Integrative Medical Sciences, Yokohama, Kanagawa 230-0045, Japan; RIKEN Center for Integrative Medical Sciences, Yokohama, Kanagawa 230-0045, Japan; Tokyo Metropolitan Institute of Medical Science, Tokyo 156-8506, Japan; RIKEN Center for Integrative Medical Sciences, Yokohama, Kanagawa 230-0045, Japan

## Abstract

The FANTOM web resource (https://fantom.gsc.riken.jp/) has been a unique resource for studying mammalian genomes, which is built on the research activities conducted in the international collaborative project FANTOM (Functional ANnoTation Of the Mammalian genome). In recent updates, we expanded annotations for long non-coding RNAs (lncRNAs) and transcribed *cis-*regulatory elements (CREs). The former was derived from the large-scale lncRNA perturbations in induced pluripotent stem cells (iPSCs) and integrative analysis of Hi-C data conducted in the sixth iteration of the project (FANTOM6). The resulting annotations of lncRNAs, according to the impact on cellular and molecular phenotypes and the potential RNA-chromatin interactions, are accessible via the interactive ZENBU-Reports framework. The latter involves a new platform, fanta.bio (https://fanta.bio/), which collects transcribed CREs identified via use of an extended dataset of CAGE profiles. The CREs, with their annotations including genetic and epigenetic information, are accessible via a dedicated interface as well as the UCSC Genome Browser Database. These updates offer enhanced opportunities to investigate the functions of non-coding regions within mammalian genomes.

## Graphical Abstract

**Graphical Abstract:**
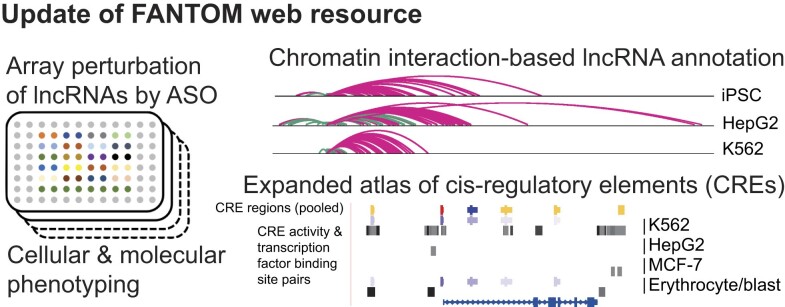

## Introduction

Understanding functional elements in the genome and their roles remains a central focus of active research. The FANTOM (Functional ANnoTation Of the Mammalian genome) consortium has long made efforts to unravel the complexity through large-scale transcriptomics and additional high-throughput experiments (1–7). The FANTOM web resource (8–13), consisting of databases and datasets that compile the results, have contributed to advancing our understanding of genome functions and have facilitated subsequent studies in the research community.

In the fifth iteration of the collaborative project (FANTOM5), we developed atlases of promoters regulating gene expression in proximity to gene transcription start sites (5), enhancers controlling gene expression from a distance (14,15), long non-coding RNAs (lncRNAs) (16), and microRNAs across multiple species (17). The core technology used in FANTOM5 was CAGE (Cap Analysis of Gene Expression), which monitors transcription start sites quantitatively across the genome at single nucleotide resolution (18–20). The sixth iteration (FANTOM6) has focused on elucidating the functions of lncRNAs with high-resolution and quantitative transcriptomics. The resulting dataset of CAGE profiles with large-scale knock-down experiments of lncRNAs in human dermal fibroblasts is available through the web resource (6).

We here present the latest updates of the FANTOM web resource, in particular, the expansion of lncRNA annotations and transcribed *cis-*regulatory elements (CREs). The former includes large-scale transcriptome data with lncRNA perturbations in induced pluripotent stem (iPS) cells (7), as well as chromatin interaction-based functional annotations of nuclear lncRNAs (21). These datasets can be explored with through the newly developed interactive framework, ZENBU-Reports (22,23). The latter provide a new set of transcribed CREs with enhanced coverage, applying a refined methodology to a broader range of experimental data. We outline the updated contents below and provide a landing page with hyperlinks to individual datasets and views: https://fantom.gsc.riken.jp/6/suppl/Nobusada_et_al_2025/.

## Results

### Functional annotation of lncRNAs in human iPS cells

Characterization of the human transcriptome revealed a large collection of lncRNAs, which are routinely transcribed with little or no protein coding potential (16,24). Although an increasing number of lncRNAs were identified in having roles in regulating multiple biological processes (25), experimentally curated functional lncRNAs represent less than 1% of all known lncRNAs (26,27). As part of the FANTOM6 project, we performed a loss-of-function genetic screen of lncRNAs in human dermal fibroblasts to experimentally identify their functionality (6); we have recently expanded this approach to human induced iPS cells (7). We employed LNA GapmeR antisense oligos (ASOs) to directly deplete RNA molecules as a strategy for perturbing lncRNAs. This approach contrasts with CRISPRi (28), which silences transcription events through epigenetic modifications. The phenotypes observed by using ASOs are likely contributed by the RNA molecule instead of by the *cis-*regulatory effect of lncRNA promoters. We conducted real time imaging and transcriptome profiling to assess cellular phenotypes and molecular phenotypes, respectively. The potential phenotypes of lncRNA extend far beyond impacts on cellular growth alone. Because of this, the phenotyping strategy employed here, is highly sensitive for identifying the functional roles of lncRNAs.

We initially selected 390 lncRNAs for ASO-based knockdown experiments and found that 200 lncRNAs showed successful knockdown by at least two ASOs. Among these, 123 lncRNAs were subjected to CAGE profiling to assess the transcriptome-level responses of the knockdowns, revealing significant molecular phenotypes for 36 lncRNAs. We identified primary *cis-*targets for 28 lncRNAs by integrating Hi-C and RADICL-seq (29) data with the differentially expressed genes (DEGs) following knockdowns. We also compared the knockdown responses of the same lncRNAs between iPS cells and human dermal fibroblast (HDF) performed previously (6). The results showed that the cellular phenotype of the same lncRNAs differed between the two cell types (Jaccard index of 5.7%), while molecular responses were more consistent. Overall, we identified lncRNAs that affect self-renewal and molecular pathways of iPS cells.

**Figure 1.**
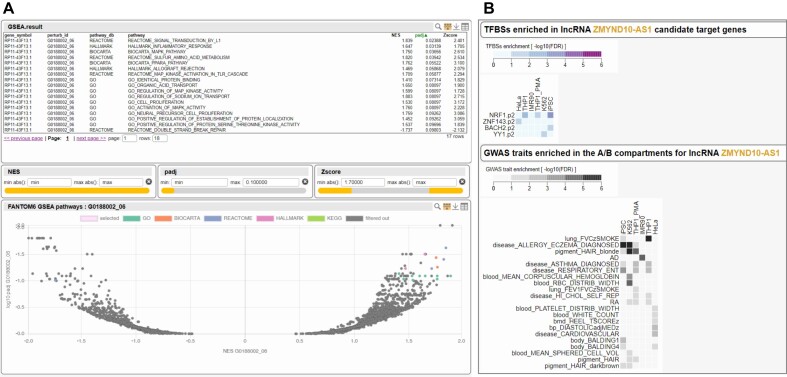
Example views of FANTOM6 lncRNA annotation interface. (**A**) GSEA results of differentially expressed genes by knock-down of ZMYND10-AS1. Users can observe which gene sets (e.g. genes in biological networks and associating with Gene Ontology terms) are affected by the knock-down in the list and volcano plot. NES: normalized enrichment score; padj: adjusted *P*-value. (**B**) Enrichment of transcription factor binding sites (TFBSs) and heritability of GWAS traits from chromatin interaction analysis of ZMYND10-AS1. By the TFBS heatmap, users can know TFBS motifs and cell types that are enriched in the target genes of the lncRNA. By the GWAS traits heatmap, users can know GWAS traits interacting with lncRNA, which can infer the function of the regulation by the lncRNA.

To facilitate effective use of these 200 lncRNA knockdown experimental datasets, we set up an interactive interface with ZENBU-Reports that integrates the analysis results (Figure 1A). Additionally, both of the raw and processed data is also available on our web site: https://fantom.gsc.riken.jp/6/datafiles/Core_FANTOM6/RELEASE_latest/.

### Chromatin interaction for nuclear lncRNA functional annotation

Most transcripts expressed in human cells do not code for proteins. While the biological functions of most lncRNAs remain unknown, some lncRNAs have been shown to have gene regulatory roles in the cell nucleus. We explored regulatory targets of lncRNAs based on chromatin interactions, as RNA-chromatin interaction data have shown that lncRNAs typically remain spatially proximal to their genomic region of interest (21). This approach may also reveal targets of lowly expressed transcripts, which are often difficult to detect with direct assay of RNA-chromatin interactions. In FANTOM6, we generated deeply sequenced Chromosome Conformation Capture Hi-C data with matched bulk, and fractionation CAGE data (cytoplasmic, nuclear and chromatin) for iPS cells to identify the target regions of nuclear lncRNAs at high resolution (6). We also reanalyzed previously published Hi-C data for 17 other human cell and tissue types using a consistent analysis pipeline, as well as RNA-chromatin interactions for iPS cell, K562, MM1S and MDA231 (21).

We identified A/B (active/inactive) compartments at 1 Mbp resolution, topologically associated domains (TADs) at 50kbp resolution, and intra-chromosomal significant genomic interactions at 10 kb resolution using Juicer (30) and GOTHiC (31). Pairwise differential Hi-C analysis was performed using an interaction count table for each replicate in each cell type or tissue. The interactions were annotated by the overlapping expressed promoters and enhancers determined using CAGE in each cell type. These annotations, including the candidate targets for each lncRNA, can be accessed using ZENBU-Reports (Figure 1B), where users can browse and compare lncRNA interactions across cell types. Raw and processed files can also be downloaded from our web site: https://fantom.gsc.riken.jp/6/datafiles/Hi-C_public_repository/.

### Interface to access the lncRNA annotations

ZENBU-Reports (22,23) is a web application that enables the creation of visual and interactive scientific portals with graphical interfaces, while providing storage and secure collaborative sharing for data uploaded by users. We used it to construct dedicated interfaces to explore each of the FANTOM6 lncRNA functional annotation efforts (See https://fantom.gsc.riken.jp/6/suppl/Nobusada_et_al_2025/), including differentially expressed genes by lncRNAs knockdown and Gene Ontology (32,33) enrichment analysis of genes interacting with a lncRNA. The interfaces allow users to browse experimental results and functional annotations of lncRNAs in tables and graphical charts (Figure 1), as well as the ability to download the provided content within the interfaces.

### Expansion of the transcribed CRE atlas – fanta.bio

In FANTOM5, we identified approximately 210 000 promoters (or CAGE peaks) and 63 000 bidirectionally transcribed enhancers in the human genome based on ∼1800 CAGE profiles (5,14,15). In the mouse genome we identified about 160 000 promoters and 44 000 enhancers based on ∼1000 CAGE profiles (Table 1). After these efforts to map CREs in the mammalian genomes, new CAGE profiles were produced as research progressed. This includes the large-scale perturbation studies conducted in FANTOM6 (6,7); the development of NET-CAGE, a method to detect enhancer RNAs sensitively through focusing on nascent RNAs (34); and additional datasets in public repositories such as SRA (35), ENA (36) and DRA (37). We decided to expand the atlas of CREs with these extended datasets.

**Table 1.** Statistics of CREs in fanta.bio and FANTOM5

Dataset		Human	Mouse
FANTOM5	CAGE profiles	1816	1018
	Promoters^a^	210 250	164 748
	Enhancers^b^	63 285	49 797
fanta.bio	CAGE profiles	6298	1264
(v1.1.0)	CREs^c^ [Non-ovalap with FANTOM5]	447 315 [352 174]	288 877 [221 249]

^a^CAGE peaks.

^b^Bidirectional pairs of CAGE peaks.

^c^Divergently transcribed peaks.

Enhancers were found as bidirectionally transcribed (38), however recent studies revealed that a subset of enhancers are unidirectionally transcribed (39,40). The approach to identify enhancers based on transcription directionality, developed in FANTOM5, may overlook those enhancers with unexpected characteristics. Thus, we developed an approach to identify CREs based on transcription divergence, a shared transcription signature between promoters and enhancers (Kawaji et al., in prep.). The pipeline identifies both promoters and enhancers simultaneously, aligning with the proposed models of transcriptional regulation in which promoters and enhancers are not mutually exclusive (41). The identified regions are classified into two classes, promoter level activity and enhancer level activity, according to their transcription intensity.

**Figure 2.**
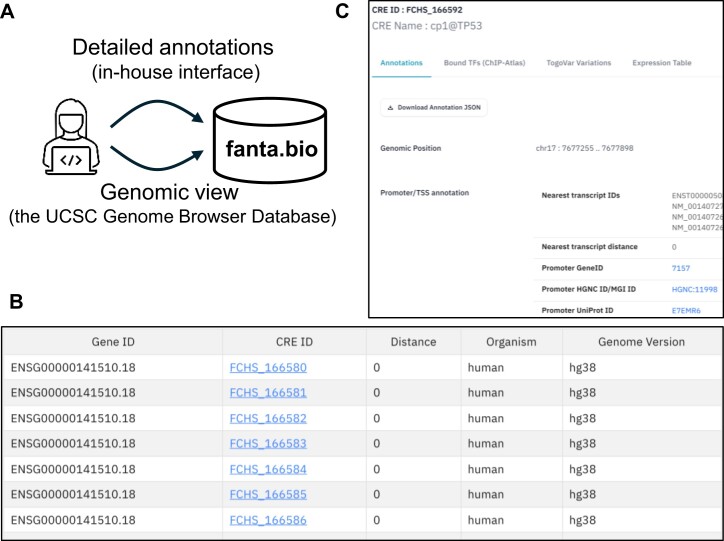
Access to and views of fanta.bio. (**A**) Two ways to access fanta.bio. (**B**) Example view of CRE search by neighboring genes (TP53) and (**C**) detailed annotation of a TP53 promoter.

We applied the method to the collected datasets, comprising CAGE profiles in human (6, 298) and mouse (1, 264), and identified 447 315 and 288 877 CREs respectively (Table 1). We compiled the results in a dedicated site fanta.bio (Functional genome ANnotations with Transcriptional Activities: https://fanta.bio/), as it contains non-FANTOM data sets, besides using FANTOM data sets as its core. We collected relevant information to interpret the CREs, such as the nearest genes, ChIP-seq peaks for transcription factors, and genome variations., In addition to providing the raw data files, we make the CREs visible through the dedicated in-house web interface as well as the UCSC Genome Browser (42) via track hub (43). (https://genome-asia.ucsc.edu/cgi-bin/hgTracks?hubUrl=https://data.fanta.bio/hub/v1.1.0-2409/trackhub/hub.txt) (Figure 2A).

The in-house web interface enables the search for CREs based on several criteria such as keywords, identifiers, neighboring genes, overlapping ChIP-seq peaks for transcription factors (TFs) processed by ChIP-Atlas (44), and proximal genome-wide association study (GWAS) SNPs curated in GWAS Catalog (45) (Figure 2B). Individual pages for each CRE provides additional information, including associated transcripts and genes for those located at promoter regions, transcription start sites provided in refTSS (46), and overlapping regions annotated as promoters and enhancers by ENCODE cCREs (47) and FANTOM5 (Figure 2C). These pages also display transcriptional activities across cell types and tissues, along with overlapping genomic variations collected in TogoVar (48) for human and MoG+ (49) for mouse.

The CREs can also be explored using the UCSC Genome Browser (50) through a track hub configuration. This setup allows for the visualization of CREs along with all genome annotations stored in the database. We provide three types of tracks: the regions of all identified CREs, their transcriptional activities in individual cell types or tissues, and ChIP-seq peaks for transcription factors processed in ChIP-Atlas. A grid-style interface with checkboxes allows for a flexible selection of tracks based on sample and track types.

### Collaborative integration with external databases

In addition to developing our own interface for accessing the datasets, we have actively collaborated with other platforms to provide broader exposure and accessibility to the datasets. The FANTOM5 datasets are now seamlessly integrated as native tracks within the UCSC Genome Browser (50) (https://genome.ucsc.edu/) for both hg38 and mm10 assemblies. Now, the researchers using the browser can access the data more efficiently with fewer steps, in comparison to the initial integration relying on track hub.

The International Human Epigenome Consortium (IHEC) Data Portal (51) provides a comprehensive set of reference epigenomes generated by IHEC, and they have recently added an ‘external hub’ function to their Data Grid view. The FANTOM5 human datasets are now accessible via the data grid view (https://epigenomesportal.ca/ihec/grid.html). The FANTOM5 human dataset can be added to IHEC data grid view by clicking on the ‘[+Add External DataHub]’ button at the bottom of the data grid.

ChIP-Atlas is a comprehensive database that aggregates a wide range of epigenomic data, including ChIP-seq, DNase-seq, ATAC-seq, and Bisulfite-seq, archived in Sequence Read Archive (SRA), and the epigenetic data is accessible through integrative genomics viewer (IGV) (52). FANTOM5 enhancers are incorporated into this resource as a part of the genome annotation suite. This allows researchers to examine these genomic regions in detail, utilizing the broad range of epigenome data.

Additionally, the metadata of the FANTOM web resources are also available in the RIKEN MetaDatabase (https://metadb.riken.jp/) (53).

## Conclusion and future updates

Since the completion of genome sequencing, numerous studies have been conducted to elucidate the diverse functions within genomes. While coding regions in genomes have been extensively studied and well characterized, non-coding regions remain less understood. In recent years, we have introduced new datasets, including CAGE profiles following a series of lncRNA knockdowns in iPS cells and integration of Hi-C data in the FANTOM6 project, to better understand the roles of lncRNAs. Furthermore, we have developed a new data platform, fanta.bio, which extends the CRE atlases established in FANTOM5. We believe that the continuous update and maintenance of the FANTOM web resource, based on the recent research advances, will facilitate further exploration of mammalian genomes in the research community.
